# Shining Light and Creating Solutions: Health and Social Needs of Older Affordable Housing Residents

**DOI:** 10.1093/geroni/igaa057.2390

**Published:** 2020-12-16

**Authors:** Lori Simon-Rusinowitz, C Daniel Mullins, Karen Morales, Rodney Elliott, Constance Raab

**Affiliations:** 1 School of Public Health, University of Maryland, College Park, Maryland, United States; 2 Pharmaceutical Health Services Research (PHSR) Department, Baltimore, Maryland, United States; 3 The PATIENTS Program, University of Maryland School of Pharmacy, Baltimore, Maryland, United States; 4 Center on Aging, College Park, Maryland, United States

## Abstract

Aging within a community requires access to health and social services. This project lays the groundwork for an innovative, three-part health and social services intervention intended to improve the health and well-being of older affordable housing residents in a low-income, vulnerable Baltimore neighborhood. We will report on the first part, an assessment of residents’ unmet health and social service needs and their ideas for meeting these needs. With guidance from a community advisory group of older residents (a key program component), we are conducting structured interviews with 50 elders to identify residents’ needs and interests. These findings will inform the next project segments: Part 2- exploring how the Village model (in which neighbors identify and offer needed services to help their neighbors age within a community) can be adapted for an affordable housing setting, and Part 3- adapting an evidence-based housing-plus-services model to meet older residents’ unmet needs.

